# Tradeoff Between Stability and Multispecificity in the Design of Promiscuous Proteins

**DOI:** 10.1371/journal.pcbi.1000627

**Published:** 2009-12-24

**Authors:** Menachem Fromer, Julia M. Shifman

**Affiliations:** 1 School of Computer Science and Engineering, The Hebrew University of Jerusalem, Jerusalem, Israel; 2 Department of Biological Chemistry, The Alexander Silberman Institute of Life Sciences, The Hebrew University of Jerusalem, Jerusalem, Israel; Massachusetts Institute of Technology, United States of America

## Abstract

Natural proteins often partake in several highly specific protein-protein interactions. They are thus subject to multiple opposing forces during evolutionary selection. To be functional, such multispecific proteins need to be stable in complex with each interaction partner, and, at the same time, to maintain affinity toward all partners. How is this multispecificity acquired through natural evolution? To answer this compelling question, we study a prototypical multispecific protein, calmodulin (CaM), which has evolved to interact with hundreds of target proteins. Starting from high-resolution structures of sixteen CaM-target complexes, we employ state-of-the-art computational methods to predict a hundred CaM sequences best suited for interaction with each individual CaM target. Then, we design CaM sequences most compatible with each possible combination of two, three, and all sixteen targets simultaneously, producing almost 70,000 low energy CaM sequences. By comparing these sequences and their energies, we gain insight into how nature has managed to find the compromise between the need for favorable interaction energies and the need for multispecificity. We observe that designing for more partners simultaneously yields CaM sequences that better match natural sequence profiles, thus emphasizing the importance of such strategies in nature. Furthermore, we show that the CaM binding interface can be nicely partitioned into positions that are critical for the affinity of all CaM-target complexes and those that are molded to provide interaction specificity. We reveal several basic categories of sequence-level tradeoffs that enable the compromise necessary for the promiscuity of this protein. We also thoroughly quantify the tradeoff between interaction energetics and multispecificity and find that facilitating seemingly competing interactions requires only a small deviation from optimal energies. We conclude that multispecific proteins have been subjected to a rigorous optimization process that has fine-tuned their sequences for interactions with a precise set of targets, thus conferring their multiple cellular functions.

## Author Summary

In nature, some proteins are more social than others, interacting with a large number of partners. These “promiscuous” proteins play key roles in cellular signaling pathways whose disruption may lead to diseases such as cancer. The amino acid sequences of such proteins must have evolved to be optimal for combined interactions with all natural partners. However, the evolutionary process leading to this promiscuity is not fully understood. We address this subject by predicting amino acid sequences that would be most compatible for interaction with each partner on its own and those most compatible for binding multiple proteins. We find that these two types of sequences are substantially different, the latter more closely resembling the natural sequences of promiscuous proteins. We also find that promiscuous proteins contain certain regions that are necessary for interfacing with all of their partners, while other regions convey specific interactions with each particular target protein. We analyze the tradeoffs required for such proteins to bind multiple partners and find that only some degree of compromise is typically needed in order to permit interactions that are seemingly antagonistic. We conclude that the simulations reported here mimic well the natural evolution of proteins that associate with multiple partners.

## Introduction

Proteins engage in numerous protein-protein interactions, which together regulate the outcome of all biological processes in the cell. By some estimates, over a third of all mammalian proteins participate in two or more highly specific protein-protein interactions [1]. Proteins that can interact with a large number of partners play a central role in the modular organization of protein interaction networks [2]. Such proteins, usually referred to as protein hubs, tend to be more essential than others for cell survival [3] and usually exhibit slower rates of evolution [4]. Moreover, the comprehensive biological activity of these proteins typically requires them to recognize a precise set of targets in a specific way. For example, each subfamily of G protein regulators interacts with only a specific subset of G proteins [5]. Proteins with diverse binding capacity have also been termed *multispecific proteins* [6],[7].

The central function of multispecific proteins within interaction networks imposes constraints on their amino acid sequences, especially in their protein-protein interfaces, i.e., the regions that are used to mediate intermolecular interactions with various targets. There exist only a few studies that have characterized in great detail the molecular and structural features of multispecific protein interfaces [8]; this is mostly due to sparse representation of such protein-protein complexes in the Protein Data Bank (PDB). A thorough understanding of atomic-level principles governing multispecific interactions is extremely important not only for the advancement of basic science but also for the design of new pharmaceuticals that modify protein-protein interactions. Furthermore, such molecular insights will provide critical feedback for systems biology research, which views protein-protein interactions from a high-level network approach [9].

**Figure 1:**
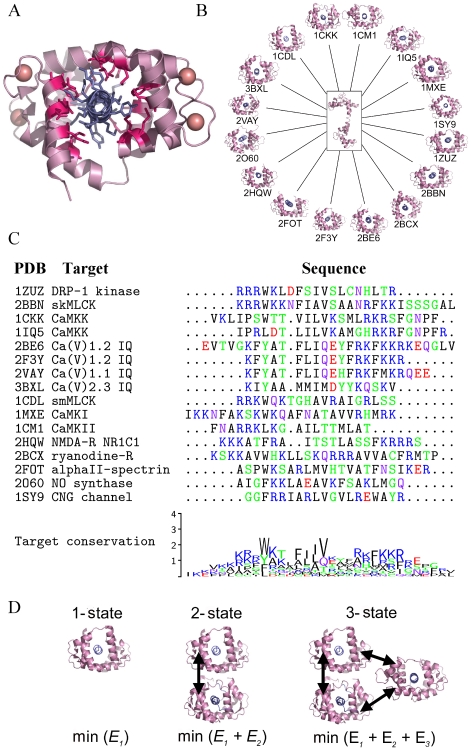
Redesigning CaM-target interactions. (A) CaM-target complex exhibiting the conventional binding mode, where CaM is shown in pink and the target peptide in violet (PDB 3BXL). The common CaM-binding interface (20 positions in total) is highlighted in magenta, and 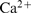 ions are indicated as pink spheres. (B) Free CaM (center) can bind each of the 16 studied targets in the binding mode shown in panel A. (C) Multiple sequence alignment (ClustalW) and conservation logo of 16 peptide targets of CaM, for each of which the solved structure shows the conventional binding mode depicted in panel A. PDB codes and target descriptions are as listed. Note that the target peptides of 2BE6 and 2F3Y are derived from the same protein; however, we used both of them since they are of different lengths and the 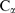 RMSD between the CaM molecules is significant (1.15 Å). (D) We methodically optimize CaM to bind each target (1-state), pairs of targets (2-state), and triplets of targets (3-state). Multiple-target design is implemented by minimizing the sum of the CaM sequence energies in each structure, with the constraint (denoted by arrows) that the same amino acid sequence be predicted for all structures.

Calmodulin (CaM) is a paradigm of a multispecific protein, with more than three hundred CaM targets identified to date [10]. CaM is the central player in the 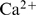 signaling pathways that control gene transcription, protein phosphorylation, nucleotide metabolism, and ion transport. This 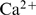 sensor protein translates the changes in 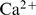 concentration into activity of many downstream targets, including kinases, phosphatases, enzymes, and ion channels [11]. Remarkably, CaM targets display considerable variability in sequence and structure. CaM-binding regions within target proteins are generally rich in hydrophobic and positively charged residues. Nevertheless, no consensus CaM-binding sequence exists for all CaM target proteins (Figure 1C). Recent structural studies have revealed that there are several binding modes accessible to CaM, allowing this protein to interact with its targets in a 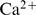-saturated state (4 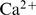 ions bound to CaM) [12],[13], in a partially-saturated 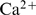 state (2 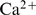 ions bound to CaM) [14], and in a 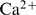-free state [15],[16]. In the 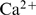-saturated form, CaM usually binds to a stretch of 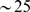 amino acids that is unfolded in the absence of CaM and becomes helical upon interaction with the protein [11]. In this “conventional” binding mode, CaM undergoes a conformational change and embraces the target helix with its two globular domains, burying a substantial hydrophobic surface area and providing favorable hydrogen bond and salt bridge interactions with the target (Figure 1A,B). 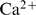-saturated CaM binds to its targets with high affinity, displaying 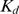 values in the 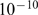 to 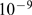 M range [17]. This affinity is reduced at least 1000-fold in the absence of 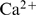, allowing for quick dissociation of CaM from its targets when 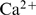 is depleted.

The multitude of binding constraints placed on CaM during evolution is likely to have produced a sequence that may not be optimal for binding to any particular CaM target, but rather presents a compromise essential for interaction with a large number of partners. In this study, we employ a computational design approach [18] to understand how the compromises required for functional promiscuity [19] are achieved both on the level of amino acid sequences and on the level of binding energetics. First, we computationally “evolve” CaM to interact with single targets; second, we evolve this protein to bind to multiple partners simultaneously. Recently, a similar analysis was performed on twenty multispecific proteins, whose interactions with two to seven targets were considered [6],[7]. In contrast to those works, we report a much more comprehensive investigation of a single multispecific protein, CaM. We examine interactions in sixteen different CaM-target complexes that exhibit the conventional binding mode. Using the structures of these complexes, we perform 697 separate CaM design calculations to obtain 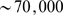 low energy CaM sequences optimal for either a single target or some combination of the targets. Rigorous quantitative and statistical comparisons of the designed CaM sequences and their energies allows us to draw conclusions regarding CaM evolution and to suggest strategies for the design of binders that are both promiscuous yet highly specific. In particular, we characterize the CaM binding interface by partitioning its residues into those that are critical for binding affinity and those that are important for multispecificity. Furthermore, we analyze the sorts of sequence compromises required to yield proteins with promiscuous interactions and show how this fits with past explanations for the ability of CaM to accommodate many targets. Finally, we examine the energetic compromises inherently crucial for multispecificity [20], and we find that our results also shed light on the unexpected findings of previous experimental protein design research.

## Results

For our study, we used all available (sixteen) high-resolution structures of CaM-target complexes that exhibit the conventional binding mode (Figure 1A,B). Note that the conformation of CaM in complex with these peptides is somewhat variant; the pairwise 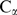 RMSD between the CaM molecules ranges from 0.84 to 7.7 Å. For each CaM-target complex, we defined the residues in the CaM binding interface. We then selected the common binding interface, a set of twenty residues, each of which interacts with the target in at least 75% of the chosen CaM-target complexes (Figure 1A). Note that, for each particular CaM-target complex, the majority of the selected residues in fact interact with the respective target (from 65–100%).

**Figure 2:**
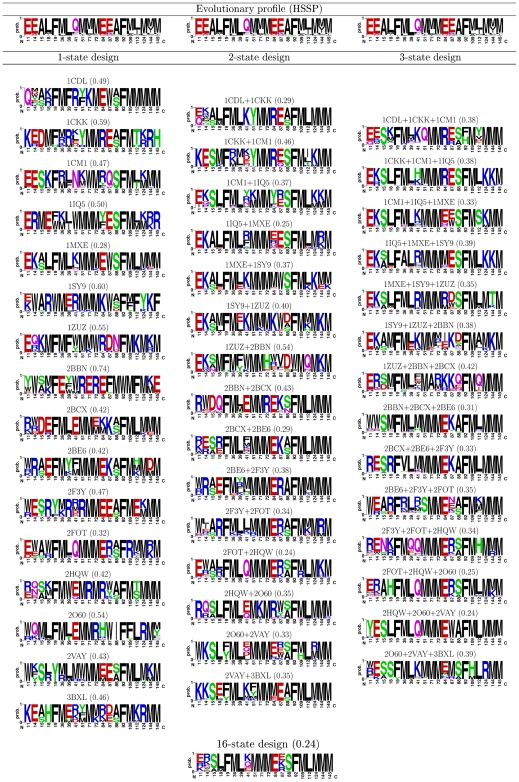
Sequence profiles for the CaM binding interface designed for interactions with one, two, three, and all sixteen targets. Amino acids found in the 100 best CaM binding interface sequences optimized for one (1-state), two (2-state), three (3-state), and sixteen (16-state) targets simultaneously, compared to the evolutionary profile of CaM (HSSP). The size of the displayed amino acid is proportional to its frequency of occurrence. Color coding: black - hydrophobic amino acids, green - polar non-charged, purple - amide, red - negatively charged, blue - positively charged. Results for all sixteen one-state CaM designs are shown. For clarity, only 15 out of 120 calculations and 14 out of 560 calculations are shown for the two-state and three-state designs, respectively. Numbers in parentheses denote the mean positional dissimilarity score (calculated according to Eq. 1) compared to HSSP, where lower values indicate greater similarity to the evolutionary profile.

Using a protein design approach, we redesigned the CaM binding interface to obtain one hundred best (lowest energy) CaM sequences for each of the sixteen selected CaM targets (“single-state” designs; see Figure 1D). In addition, a hundred best CaM sequences were designed for all possible sets of two and three targets simultaneously, resulting in 120 and 560 separate calculations (“two- and three-state” designs). Consideration of more than three CaM targets in a combinatorial manner is computationally prohibitive. Thus, we next proceeded to design a hundred sequences best suited for binding all sixteen targets. In total, we performed calculations for almost 700 design scenarios (Figure 2) and predicted 100 sequences for each scenario. The CaM sequences were designed with an atomic-level energy function that included van der Waals, electrostatic, hydrogen bonding interactions, and a surface-area-based solvation term [21]. To overcome the high combinatorial complexity of the design calculations, we utilized a number of search algorithms in parallel to obtain the lowest energy CaM sequences: the first is based on the dead-end elimination (DEE) theorem [22], the second is based on belief propagation (BP) for probabilistic graphical models [7], and the third was Monte Carlo simulated annealing [23] (only for the 16-state design); see Methods for details. The results from the various methods were combined to compile a list of the hundred best CaM sequences designed for a particular scenario. These hundred sequences were used to calculate amino acid occurrence frequencies at each CaM interface position (Figure 2).

To analyze the design results, we computed the evolutionary profile for the residues belonging to the CaM binding interface using the Homology-derived Secondary Structure of Proteins (HSSP) database (Figure 2 , top). The CaM HSSP profile (henceforth referred to as the evolutionary profile) revealed that the interface is highly conserved through evolution and is composed of predominantly hydrophobic amino acids supplemented by a few glutamates and a single glutamine. Surprisingly, the defined interface has a pseudo two-fold symmetry, where the same motif is utilized for target recognition in the CaM N- and C-terminal domains (EEAFMLMMM), with the addition of L18 and Q41 in the N-terminal domain.

### Similarity between the designed and native CaM sequences

**Figure 3:**
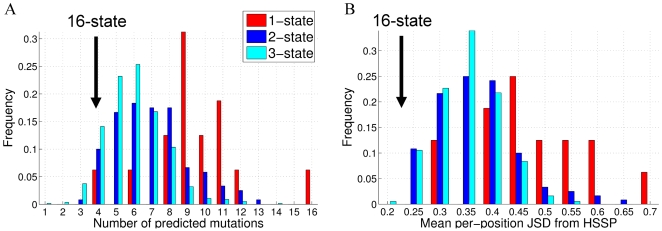
Comparison of the lowest energy CaM sequences designed for one, two, or three targets with native and evolution-derived CaM sequences. (A) The number of mutations from the WT CaM sequence observed in the single lowest energy sequence when redesigning 20 positions in the CaM binding interface. The average number of mutations is 9.5 for single-state designs (52% native sequence recovery), 7 for the two-state designs (65% recovery), 6 for the three-state designs (70% recovery), and 4 for the design of all sixteen states (80% recovery). (B) Comparison of the 100 best designed CaM sequences to the evolutionarily observed sequences (HSSP profile of CaM). JSD dissimilarity between distributions of 100 designed sequences and HSSP was calculated according to Eq. 1, where lower JSD values indicate greater similarity between the predicted sequence profile and the HSSP profile. The average JSD is 0.48 for single-state designs, 0.37 for two-state designs, 0.35 for three-state designs, and 0.24 for design of all sixteen states.

First, we assessed the similarity of our designed CaM interface sequences to the native CaM sequences. The number of mutations predicted in the single best CaM sequence, designed for interaction with one target, ranges from four to sixteen with a mean value of 9.5 (Figure 3A). When two CaM targets are included in the design, the number of predicted mutations ranges from 3 to 13 with a mean of 7 mutations. The distribution of predicted mutations further shifts to the left when three CaM targets are incorporated into the design, exhibiting a mean of 6 mutations. Incorporation of all sixteen states in the design procedure resulted in only 4 mutations. Next, we compared the distribution of amino acids obtained from the one hundred CaM sequences designed for interactions with one, two, and three targets. This was done by calculating the Jensen-Shannon divergence (JSD, see Methods) between the evolutionary profile of CaM and the amino acid distribution obtained after CaM redesign. A JSD score of 0 corresponds to identical distributions, while a JSD score of 1 corresponds to completely discordant distributions. We henceforth refer to the JSD score as the “dissimilarity score”. A comparison of the hundred CaM interface sequences designed for one, two, and three targets (Figure 3B) showed the same trend as observed for the single best CaM sequences. The highest dissimilarity scores were obtained for single-state designs (mean value of 0.48), medium scores were obtained for two-state designs (mean value of 0.37), lower scores were obtained for three-state designs (mean value of 0.35), and the lowest score was obtained for sixteen-state design (0.24).

### Analysis of the single-state design scenarios

**Figure 4:**
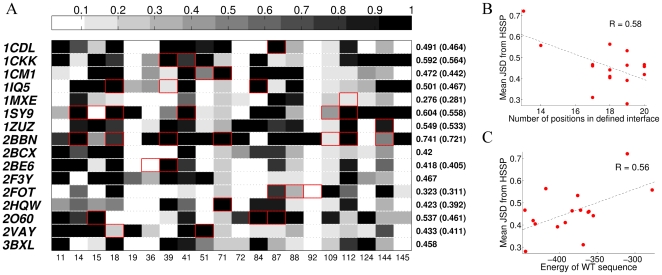
Sequence comparison of single-state CaM designs and the evolutionary profile of CaM. (A) For each position in the CaM binding interface (horizontal axis), dissimilarity with the evolutionary profile of CaM (HSSP) is calculated using the JSD score. Black - positions with the largest dissimilarity between the design and the HSSP. White - positions showing the largest similarity between the design and HSSP. Red boxes indicate positions that are not in the binding interface for a particular CaM-target complex but were included in the calculation as part of the common binding interface. On the right, the average per-position dissimilarity is given for the 20 interface positions in the particular CaM-target complex. In parentheses, the same number is calculated with the boxed (non-relevant) interface positions excluded, so that the dissimilarity tends to decrease for these more “relevant” positions. (B) Correlation between the number of relevant interface positions in a particular CaM-target complex structure and dissimilarity of the designed sequences with the evolutionary profile, as calculated by the mean per-position JSD score (right side of panel A, numbers in parentheses). (C) Correlation between the energy of the WT sequence threaded onto a particular CaM-target complex structure and dissimilarity of the designed sequences with the evolutionary profile, as calculated by the mean per-position JSD score (right side of panel A).

We next compared the hundred best CaM sequences designed for interactions with the various single targets. For each of the interface positions, we calculated the dissimilarity score between the distribution of designed amino acids and the evolutionarily-derived distribution (Figure 4A). Our analysis revealed that, at some of the CaM interface positions, our design calculations predicted a distribution very similar to the evolutionary profile for the majority of the CaM-target complexes (columns with lighter boxes, Figure 4A). On the other hand, at other positions, the design methods predicted amino acid distributions very different from the evolutionary profile (columns with darker boxes). Among the 16 different CaM-target complexes, the average per-position dissimilarity score was very diverse and ranged from 0.276 to 0.741 (mean of 0.48), so that some structures inherently predict profiles much more similar to the evolutionary profile than others. These scores slightly decreased (numbers in parentheses) if we excluded from our analysis the CaM positions that belong to the common CaM binding interface but do not interact with the target in the particular CaM-target complex. We also noticed that the designed CaM sequences are more similar to the evolutionarily-defined CaM sequences for the targets that interact with a larger number of CaM residues. Figure 4B shows that there is an inverse correlation (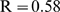) between the dissimilarity with the evolutionary profile and the number of the designed CaM positions that are in the binding interface for a particular CaM-target complex. In addition, not unexpectedly, the designed CaM sequences come out somewhat more similar to the native profile if the WT CaM sequence is predicted to be strongly compatible with the CaM-target complex structure. This is demonstrated in Figure 4C, which shows a correlation (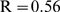) between the dissimilarity with the evolutionary profile and the energy of the WT CaM sequence in the context of a particular structure.

**Figure 5:**
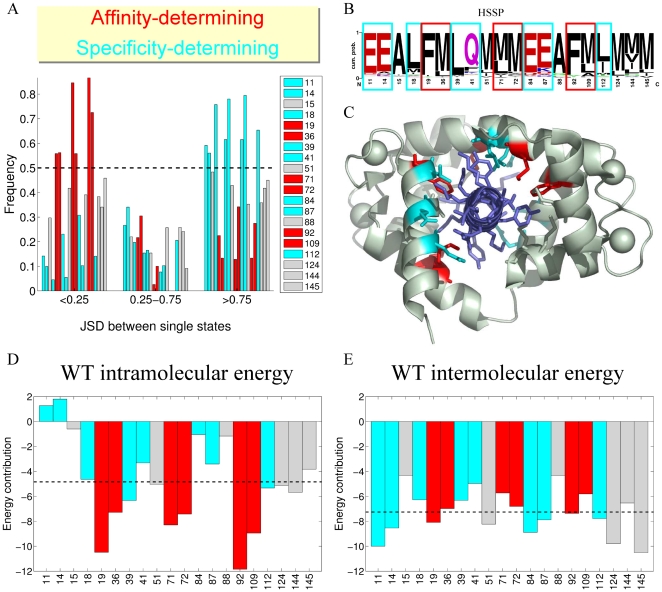
Prediction of affinity- vs. specificity-determining positions. (A) Dissimilarity between all pairs of sequence profiles designed for a single structural state was calculated for each of the interface position by computing the JSD dissimilarity score (Eq. 1). The results were binned for histogram analysis. Positions that exhibit low pairwise JSD scores with higher frequency (red) are most conserved between the various CaM single-state designs and hence are predicted to be affinity-defining. Positions that exhibit high pairwise JSD with higher frequency (cyan) differ for each single-state design and hence are specificity-defining. (B) Evolutionary logo with specificity and affinity-defining positions marked. (C) Structure of a CaM-target complex (PDB 3BXL) with affinity and specificity positions marked in red and cyan, respectively, and the target peptide is colored in violet. (D) Intramolecular and (E) intermolecular energetic contributions for the WT CaM sequence at each of the 20 interface positions. The intra- and intermolecular contributions were calculated in each of the 16 CaM-target complexes and were averaged over all cases. Positions are colored as above, and the dotted line indicates the average energy contribution for all positions.

Next, we quantified the correlation among the hundred best sequences designed for interactions with different single targets. This was done by calculating the dissimilarity score between all possible pairs of single-state designs at each of the design positions. This type of analysis allowed us to identify the CaM binding interface positions that, on the whole, exhibit similar amino acid identities in all CaM-target complexes (affinity-defining positions: 19, 36, 71, 72, 92, 109) and the positions that display much greater diversity among the single-state designs (specificity-defining positions: 11, 14, 18, 39, 41, 84, 87, 112) (Figure 5A). In the evolutionary profile of CaM, the affinity-defining positions are occupied by hydrophobic residues, either Met, Leu, or Phe. The specificity-defining positions, on the other hand, are dominated by hydrophilic amino acids (Glu and Gln) and, in some cases, are occupied by Leu (Figure 5B). The affinity- and specificity-defining positions are present in both the N- and the C-terminal domains of CaM and are also distributed evenly throughout the CaM structure (Figure 5C). In addition, we could not detect any differential pattern in the way the targets interact with either class of CaM positions (since the CaM targets do not exhibit distinctly conserved motifs; see Figure 1C).

In an attempt to further understand the differences between interactions defining affinity and specificity in native CaM, we threaded the WT CaM sequence onto all sixteen selected CaM-target complexes and calculated the energetic contribution of each of the binding interface positions to the total energy. The energetic contributions at each position were further separated into intra- and intermolecular energies, corresponding to stabilization within CaM and between CaM and the target, respectively. We further averaged the per-position energetic contributions for the sixteen CaM-target complexes. We saw that there is a distinct difference in how the affinity- and specificity-defining positions stabilize the WT CaM-target complexes. This difference is especially striking for the intramolecular energy contributions (Figure 5D). The six affinity-determining positions exhibit the highest intramolecular contributions among all positions, being crucial for stabilization of CaM in the target-bound conformation. The majority of the specificity-determining positions, on the contrary, exhibit higher than average, and sometimes even unfavorable, contributions to the intramolecular energy. However, most of these specificity-determining positions contribute more than average to the intermolecular energies, being more important for direct interactions with the target (Figure 5E).

**Figure 6:**
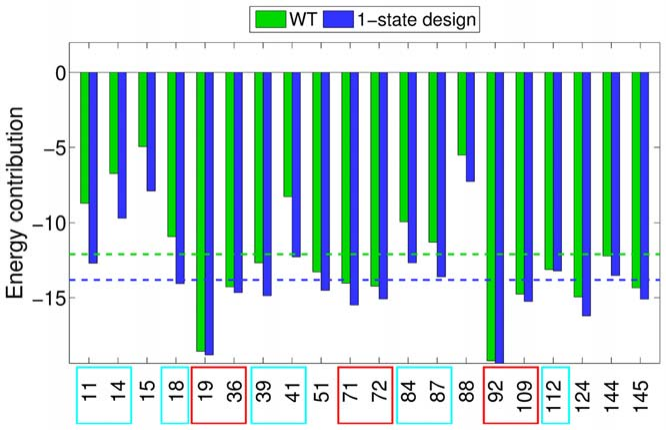
Comparison of per-position energies between WT and single-state design sequences. Total energies (intramolecular+intermolecular energies) for each of the 20 interface positions are averaged in all 16 structures for the native sequence (green bars) and the single-state design lowest energy sequences (blue bars). The dotted lines indicate the respective average energy contributions for all positions. The affinity- and specificity-determining positions are boxed in red and cyan, respectively.

We next investigated what happens to the energetic contributions in the CaM sequences designed for interactions with the single targets (Figure 6). This was done by computing the total energy contribution of each designed position first for the single best designed sequence and then for the WT CaM sequence, for each of the sixteen CaM-target complexes. The per-position energetic contributions were then averaged over the sixteen cases. Figure 6 shows that, at all design positions, the energetic contribution is either unchanged or is improved for the designed sequences compared to that of the WT CaM sequence. An unchanged value is observed at positions that are highly optimized for interaction with the target, including most of the affinity-defining positions. Large improvements in the energetic contributions from the WT to design are observed for positions where the WT energies were less favorable, including the majority of the specificity-defining positions.

### Sequence comparison of single-state and two-state designs

**Figure 7:**
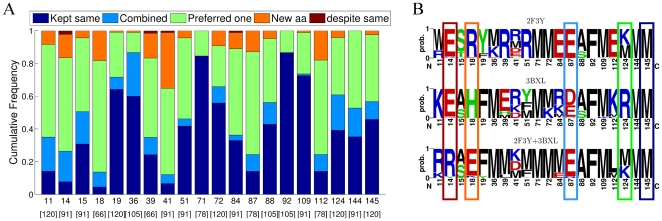
Categories of multistate sequence compromise. (A) In comparing the amino acid distributions at each of the CaM interface positions obtained in single-state designs with those resulting from 2-state design calculations, five scenarios were observed. Dark blue - both individual states have similar profiles and the 2-state design chooses this profile. Light blue - two-state design yielded a profile that is a combination of the two distributions obtained for each single-state design. Green - two-state design yielded a distribution of amino acids that was similar to that of only one of the single-state designs. Orange - an amino acid distribution for the two-state design was chosen that is different from that of both of the individual single-state designs. Maroon - Despite the individual states having similar profiles, the two-state profile is different. Interface positions are marked on the horizontal axis. The analysis was performed only for cases where the particular position is in the binding interface for both of the combined CaM-target complexes (the number of such cases is shown in brackets below the interface position number). (B) Logos of sequence profiles individually optimized in the context of CaM-target complex structures with PDB identifiers 2F3Y and 3BXL (1-state design), compared to the profile resulting from simultaneous optimization for interaction with both targets (2-state design). Positions that demonstrate compromise scenarios are outlined in colors as in panel A.

CaM needs to achieve a certain compromise to obtain a sequence compatible with binding each of the two targets. Comparison of the CaM sequences designed for interactions with each of the two single targets (single-state designs) and the combinations of these two targets (two-state designs) revealed that the compromise could be achieved via five different scenarios. This is demonstrated in Figure 7 using the examples of CaM-target complexes, corresponding to the PDB codes 2F3Y and 3BXL. In the most trivial scenario, CaM sequence profiles designed for the two single targets have an identical or very similar amino acid distribution at a particular position (e.g., position 145 in Figure 7B). This amino acid distribution remains the same when CaM is designed to interact with both of these targets (“Kept same” in Figure 7A). In the second scenario, two different amino acid distributions are observed for the single-state designs. However, the sequence profile designed for both targets is similar to both of the two distributions resulting from the single-state designs, since it combines them in some form (“Combined” in Figure 7A and e.g., position 87 in Figure 7B). In the third scenario, two different amino acid distributions are observed for the single-state designs, while in the two-state design, one of these distributions dominates (“Preferred one” in Figure 7A and position 124 in Figure 7B). In the fourth scenario, two different amino acid distributions are again observed for the single-state designs. In the two-state design, however, a new amino acid distribution appears; this distribution is significantly different from those observed for both single-state designs (“New aa” in Figure 7A , position 18 in Figure 7B). In the fifth scenario, an identical amino acid distribution is observed for the single-state designs. Interestingly, a new amino acid distribution appears in the two-state design (“despite same” in Figure 7A , position 14 in Figure 7B). This scenario, however, occurs only very infrequently throughout our design calculations. Expectedly, the affinity-determining positions in CaM (19, 36, 71, 72, 92, 109) tend to exhibit the “Kept same” category of compromise, while the specificity-determining positions (11, 14, 18, 39, 41, 84, 87, 112) tend to select the “Preferred one” category.

**Figure 8:**
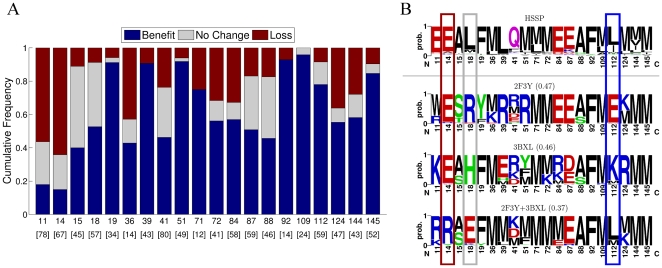
Effect of multispecific compromise on the similarity of the designed sequences with the evolutionary profile. (A) For each position in all 2-state designs, its dissimilarity with the evolutionary profile (JSD score with HSSP) is compared to the JSD dissimilarity with the distribution that is the average of the two respective 1-state design strategies (JSD score for 1-state design results). For each position, we only analyzed the scenarios in which the predicted profile preferred only one of its constituent states or contained novel amino acids (the number of such cases for each position is shown in brackets on the bottom). Three main outcomes were tallied: Gray - no significant change in the dissimilarity score. Blue - the two-state design significantly improves the JSD of the single-state designs, i.e., two-state design is beneficial. Red - the two-state design performs significantly less well than the single-state designs in recovering the HSSP profile, i.e., two-state design results in greater dissimilarity with the evolutionary profile. Interface positions are marked on the horizontal axis. (B) Logos of sequence profiles individually optimized for CaM interaction states 2F3Y and 3BXL (1-state design) and simultaneously optimized for both states (2-state design), compared to the evolutionary profile (HSSP). Mean dissimilarities with the evolutionary profile (JSD from HSSP) are noted in parentheses. Positions that demonstrate the effect of the multispecific compromise, vis-a-vis HSSP, are outlined in colors as in panel A.

We next investigated if the compromises required to achieve multispecificity bring the CaM sequence closer to its evolutionarily-derived sequence profile. For this purpose, we compared the amino acid distributions resulting from the single-state designs with those from the two-state designs, all in relation to the evolutionary profile (Figure 8). Here, we discarded the scenarios where the two-state design produced results similar to both single-state designs (“Kept same”, “Combined”), since these scenarios do not result in changes relative to the evolutionary profile of CaM. Interestingly, for most of the designed positions, CaM sequences optimized for two targets were more similar to the evolutionary profile than those optimized for single targets (“Benefit” in Figure 8A and position 112 in Figure 8B). In a few cases, no significant change was observed vis-a-vis the evolutionary profile (“No Change” in Figure 8A , position 18 in Figure 8B), while in some cases the amino acid distribution becomes more different from the evolutionary profile compared to that of the single-state designs (“Loss” in Figure 8A , position 14 in Figure 8B).

**Figure 9:**
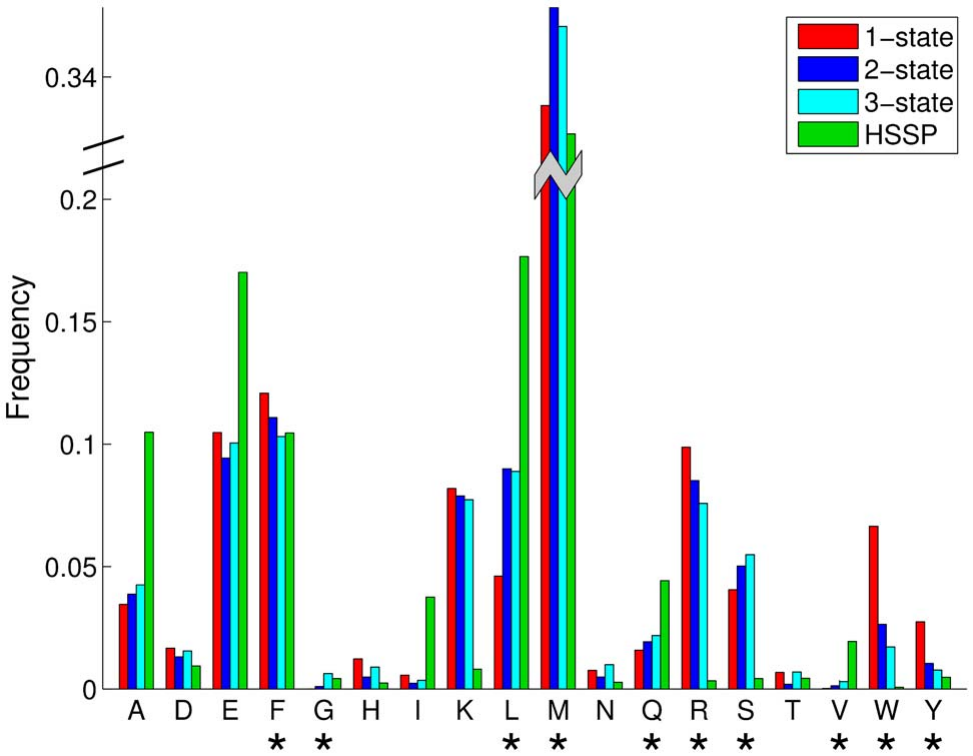
Amino acid composition of CaM interface designed for one, two, and three targets and that designed by evolution. Asterisks mark those amino acids with frequencies that significantly differ (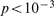, t-test with Bonferroni correction) between 1-state and both multistate designs, and change monotonically from 1-state to 2-state to 3-state (within a threshold of 90%).

It is interesting to see how the overall amino acid composition (calculated for all 100 best sequences) changes from the CaM interface sequences designed for interaction with a single target to the sequences designed for multispecificity (either two-state or three-state design). Figure 9 shows several significant differences between the two situations. Methionine dominates the compositions of the CaM-binding interface for single-state designs. They become even more frequent when CaM is designed for interactions with two or three targets. In addition, we noted a significant increase in the number of Leu, Gln, Ser, Gly, and Val when introducing additional interaction constraints on the CaM sequence. On the other hand, all aromatic amino acids (Phe, Trp, Tyr), as well as Arg, become significantly less abundant when more than one CaM target is considered in the design.

### Energetics of the designed CaM-target interactions

**Figure 10:**
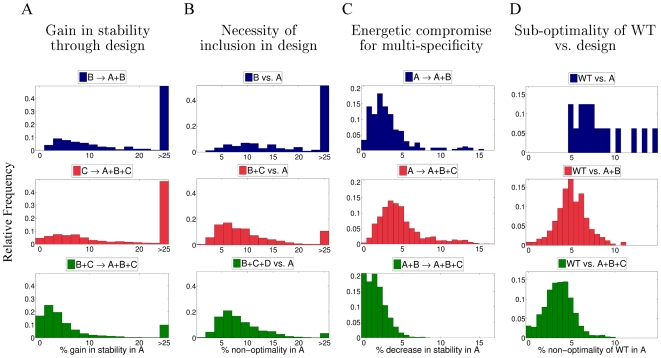
Energetic compromise of the designed and WT interface sequences due to introduction of multispecificity. We evaluate the compatibility of various sequences with the structure of CaM in complex with target A (denoted as “state A”), where the choice of A ranges over all sixteen CaM-target complex structures; B, C, and D denote the structures of other CaM-target complexes. The sequence energies compared in the context of CaM in complex with A are those predicted by our protocol while considering CaM interactions with various combinations of targets, e.g., A+B. Each plot is a histogram of changes in energy resulting from the comparison between such design scenarios. All energy differences are normalized relative to the lowest energy sequence designed for interactions with target A and capped at 25% for purposes of depiction. (A) Gain in stability of state A due to its incorporation in multispecific design. Top: 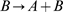 indicates that energies in state A were compared between the sequences resulting from CaM design that considers only interactions with target B and the design that simultaneously considers interactions with A and B. Middle: 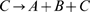 compares the sequences designed for state C and those designed for states A+B+C. Bottom: 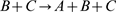 compares those designed for B+C with those designed for A+B+C. (B) Energetic non-optimality of state A *not* included in a particular multispecific design scenario. The energy differences are calculated between sequences designed for interactions with the marked combination of targets (B, B+C, and B+C+D, respectively) and those designed only for interaction with target A. (C) Loss of stability of state A due to incorporation of additional states in the design. Top: 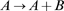 compares the energies of the sequences designed for interaction with target A alone with those designed for both A and B simultaneously. Middle: 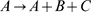 compares the sequences designed for A with those designed for A, B, and C. Bottom: 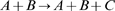 compares the sequences designed for both states A and B with those designed for A, B, and C. (D) Energetic non-optimality of the WT CaM sequence in state A, as compared to the lowest energy sequence predicted in the respective design scenario, including designing only for interactions with A (top), for interactions with A and B (middle), and for interactions with A, B, and C (bottom).

In this study, we designed 100 CaM binding interface sequences for each of 697 design scenarios (1-state, 2-state, 3-state, and 16-state). We computed the energy of each of these sequences in the context of all sixteen structures of the CaM-target complexes. Each design scenario was assigned an energy value in each structure; this energy value was the minimum of the energies obtained by the 100 sequences designed in this scenario. We next analyzed how these energies vary as additional targets are either introduced into, or removed from, the design procedure (Figure 10). Note that the frequency histograms in Figure 10 are based on up to 7000 comparisons of energies between design scenarios.

We denote by A, B, C, and D any four arbitrary CaM states, i.e., complexes of CaM with different targets. Firstly, we asked how incorporating additional CaM-target interactions affects the stability of the newly incorporated CaM-target complex, as opposed to performing the same design without this complex (Figure 10A). For example, 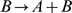 denotes that energies in state A were compared for the sequence resulting from design in state B and the simultaneous design in states A and B (A+B). As expected, adding a state (A) to the design procedure, when already designing for a different state (B), yields a significant increase in the stability of the designed CaM sequence in state A (with 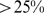 increase in stability for almost half of all such cases). Similar gains in the stability of a newly incorporated state (A) were observed in the transition from one state (C) to a total of three states (A+B+C, middle panel of Figure 10A). On the other hand, when already designing for two states (B+C), incorporating an additional state A (A+B+C) yielded much lower gains in stability for that state (leftward shifted distribution, bottom panel). This is due to the fact that performing 2-state design for B+C already predicts a sequence somewhat compatible with A (middle panel of Figure 10B).

Next, we examined the necessity of actually including a particular state in the design process (Figure 10B). For example, if two CaM-target interactions were very similar in nature (due to relatedness of the targets), then simply designing for one of these interactions would suffice in stabilizing the other. We did not find this to be the case for our sixteen targets, as designing for one state (B) results in sequences that are highly unstable in state A (B vs. A). Such sequences are 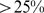 sub-optimal for almost half of all cases (top panel). However, designing for two states (B+C) or three states (B+C+D) yields sequences that are significantly more compatible with the binding of target A (middle and bottom panels).

Thirdly, we investigated the effect of incorporating other states into multispecific design on those states that are already included in the design process (Figure 10C). Expectedly, we found that incorporating an additional state (B) into the design process (top panel) resulted in CaM sequences that are less optimized for interaction with the first target (A). Incorporating two additional states (B+C) yielded sequences with an additional decrease in stability when interacting with target A. On the other hand, when already designing for two states (A+B) and adding a third state (A+B+C), the resulting CaM sequences exhibit a smaller decrease in optimality for target A. Thus, overall, we found that a large decrease in stability occurs when incorporating one additional state, but adding a third state does not have the same effect (top vs. bottom panels).

Finally, since the WT sequence is optimized to bind all sixteen targets studied here, we expected it to posses sub-optimal stability in the complex with any particular single target. Indeed, our analysis showed that the WT CaM sequence, when threaded onto the structures of all sixteen CaM-target complexes, always obtains a substantially higher energy compared to that of sequences optimized for these structures (Figure 10D , top panel). Note that a related phenomenon was also observed above for individual design positions (Figure 6). However, the relative sub-optimality of the WT sequence in a particular interaction (with target A) progressively decreases when compared to sequences optimized for interactions with two targets (A+B, middle panel) and three targets (A+B+C, bottom panel). Thus, WT sequences seem to be most energetically similar to sequences optimized for multispecificity.

## Discussion

### How good are the designed CaM sequences?

The CaM interface sequences that we designed to best interact with single targets have an average of 9.5 mutations, corresponding to a 52.5% wild-type recovery rate (Figure 3A). Our WT recovery rates for single-state CaM designs are very similar to those observed, on average, when redesigning protein cores (51%) [24] and somewhat lower than that observed in our previous study, where the interface of a very high-affinity protein-protein complex was redesigned (62%) [25]. These results are reasonable, since CaM interactions with its targets are mostly conveyed by buried residues; the affinities of CaM-target complexes, while high, are not among the highest measured in nature. On the other hand, our WT recovery rates for single-state designs are considerably higher than those observed by Humphris et al. when redesigning the interfaces of twenty multispecific protein-protein complexes [6]. In many of their examples, however, a significant fraction of the redesigned positions do not interact with the target in each particular protein complex under design and are thus likely to mutate without any constraints. Moreover, we demonstrated that the WT recovery rate for design of the CaM interface is proportional to the number of residues directly interacting with the target (Figure 4B). Having more interface residues results in the addition of intermolecular contacts to the network of molecular interactions [26], better reproducing the environment within the native CaM interface. Hence, our higher WT recovery rates for single-state CaM designs, as compared to those reported by Humphris et al., are easily explained by the high fraction of the designed CaM positions being found in direct interaction with the target for each CaM-target complex considered (85% or more for all but 2 of the complexes). Interestingly, CaM interface sequences designed using NMR structures as templates gave significantly higher dissimilarity scores with the CaM evolutionary profile (2BBN and 1SY9 in Figure 4A) than those sequences obtained using X-ray structures as templates (all others); note that these structures also have the fewest of the commonly defined interface positions interacting with their respective targets. The lower rates of native sequence recovery in design calculations using NMR structures imply that these structures may be less optimal templates for protein design calculations, in agreement with recent findings by Schneider et al.[27].

When optimizing the CaM binding interface for two, three, or sixteen targets simultaneously, our WT sequence recovery rate increases from an average of 52% to an average of 65%, 70%, and 80%, respectively. These WT recovery rates are similar to those observed previously when redesigning multispecific proteins by considering several partners together [6]. Our high-level sequence analysis of the design predictions demonstrates that the native CaM binding interface sequence is not optimal for interaction with each target on its own but fits well the multispecific requirements imposed by nature. Moreover, our novel design procedure, which includes progressive incorporation of additional targets into the design, provides a plausible scheme for CaM evolution in nature. Specifically, when designing CaM to possess binding affinity to all 16 targets studied here, the predicted interface sequence is quite similar to that resulting from evolution (Figure 3). In theory, we expect the WT recovery rate for the CaM binding interface sequence to approach 100% if all native CaM targets were taken into account. Deviation from this number would result from inaccuracies in the energy function used for design (see below), or possibly from other constraints that this technique does not currently incorporate, e.g., sequence composition preferences for the organism.

When evaluating our designed CaM interface sequences, we noticed that many of these sequences are more positively charged than the evolutionary profile of CaM (Figure 9). This increase in positive charge on the CaM interaction surface could, in principle, bring about a reduction in affinity between the redesigned CaM and its targets. Nonetheless, our previous experimental studies of CaM interactions with two separate targets revealed that carefully designed charge-reversal mutations in the CaM binding interface do not reduce CaM affinity to targets and, in some cases, even increase the affinity [17],[28]. In addition, these charge-reversal mutations help to increase CaM binding specificity [28]. Still, it is also possible that our design calculations are slightly biased toward incorporating Lys and Arg residues, which have many atoms to participate in more interactions and a larger number of rotamers; hence, they may be chosen more often than other amino acids.

The energy function and molecular models we used for CaM design might not realistically portray all atomic interactions, although they have been experimentally verified for many cases, e.g., [17],[21]. It has recently been pointed out that some inaccuracies in energy functions can be overcome by averaging the results of many protein design calculations [29]. In this work, we tried to minimize the effect of possible errors by designing 100 sequences compatible with each design scenario and by averaging the results obtained from all possible combinations of two- and three-state CaM designs. Additional sources of modeling errors include the use of both a fixed protein backbone and rigid amino acid side chains (rotamers). Some contemporary research has attempted to overcome these limitations by permitting the backbone to be flexible [30]–[33], the side chains to move more continuously [34], or both [35]; however, introduction of additional flexibility is computationally expensive and hence would be incompatible with our high-complexity 700 design scenarios. In short, while our calculations could be inaccurate in some particular instances, overall they fit well with similar computational and experimental work and should be reliable in predicting general and unbiased trends in CaM evolution.

### Lessons on the evolution of multispecific proteins

The per-position analysis of amino acid compromises required for achieving multispecificity in CaM followed several scenarios, two of which are especially interesting (Figure 7A). In the first situation, a new amino acid appears in the two-state design that is different from amino acids observed in both single-state designs (“New aa”). This amino acid, while not optimal for interaction with each target on its own, was predicted to be the best compromise satisfying interactions with both targets. Interestingly, in the majority of cases where such a scenario was observed, the new amino acid was more similar to the evolutionary profile of CaM (e.g., position 112 in Figure 8B). This scenario demonstrates how the native CaM sequence has acquired its identity. In another interesting (but rare) scenario, we observed that the amino acid distribution in the two-state CaM design was different from that in both single-state designs in spite of the latter distributions being identical (“despite same”, position 14 in Figure 7B). This scenario is likely to be due to correlated mutations. For example, positions 14 and 18 in Figure 7B are coupled to each other. Thus, in spite of the fact that Glu dominated position 14 in both single-state designs, the appearance of Glu18 in the two-state design forces the appearance of Arg at position 14.

In this work, we classified the CaM binding interface residues as either affinity- or specificity-defining [36],[37]. Our predictions were derived solely from sequence comparisons, with affinity-determining residues being very similar to each other among all single-state designs and specificity-determining residues differing the most. Previous studies found that the residues that maximally contribute to protein-protein interactions (hot-spots) are also more evolutionarily conserved [38] and tend to be grouped into spatially distinct clusters with strong interactions within the clusters [39],[40]. In agreement with these findings, the CaM interface positions that are most “conserved” among the designs (affinity-determining) are also very stabilizing for the native CaM-target complexes, and these six “hot-spot” positions are clustered into three pairs (19 and 36; 71 and 72; 92 and 109; see Figure 5C). Unexpectedly, the strong energetic contributions of the hot-spot residues were largely mediated by intramolecular interactions (Figure 5D), meaning that the affinity-defining residues in CaM mostly stabilize it in the target-bound conformation. On the other hand, the specificity-determining residues often have an unfavorable effect on CaM intramolecular energies but provide favorable interactions with each particular target (Figure 5E). Thus, the coupling between evolution and energetics is very strong in CaM, and the pattern of this coupling can even be used to infer that large conformational changes accompany target recognition by CaM. This finding is consistent with the population shift model [41]–[44], which asserts that an unbound protein samples a multitude of conformations; the equilibrium is shifted towards the bound state upon addition of the binding partner. Our results suggest that the affinity-determining positions enable the transition to each of the bound CaM states, while the specificity-determining positions lock CaM into a target-specific conformation. We postulate that an analogous scenario should be detected for other multispecific proteins that undergo conformational changes upon binding. Finally, we also validated our positional classifications using the INTREPID web server for predicting functionally important residues (based on evolutionary sequence conservation) [45]. For the 142 CaM positions, the 6 affinity-determining residues were among the 14 ranked most important for function, while the 8 specificity-determining residues were ranked significantly lower than average. The latter is not unexpected, since these positions convey distinct favorable interactions with various targets and are hence not conserved at higher levels in the evolutionary hierarchy (not shown).

The energetic analysis of the WT and designed sequences in the context of all sixteen structures revealed a few interesting conclusions. Firstly, we demonstrate that, from an energetic perspective, the CaM interface is optimized for binding multiple partners but sub-optimal for interaction with each particular target (Figure 10D , top vs. middle and bottom). This result is in accord with previous studies, which have shown that binding promiscuity results in weaker affinity toward targets [46]. Additionally, we find that designing the CaM interface for additional functions requires a notable tradeoff in stability that escalates as more functions are simultaneously added (Figure 10C , top and middle). This finding is consistent with conclusions from mutational studies of enzymes, where function-stability tradeoffs were observed in positions that are highly constrained by the catalytic mechanism [19],[20]. Nevertheless, the loss of binding stability associated with acquiring a second binding partner is only minor when balancing it with the huge gain in CaM's favorable interactions with this new target (Figure 10C vs. Figure 10A). Finally, it is of great interest that, when gaining the ability to bind a third partner, the energetic penalty imposed on the interactions of CaM with its original two partners is not that great (Figure 10C , bottom vs. top). This could explain why the transition from three-state to sixteen-state designs does not bring about a very large difference in predicted mutations (Figure 3). Furthermore, these results would suggest that the evolution of multispecific proteins may be subject to a phenomenon of positive feedback, where once a protein becomes somewhat promiscuous, it can be virtually uninhibited in the expansion of binding partners similar to the ones it already binds [47]. This phenomenon could partially contribute to the high connectivities of hub proteins (such as CaM), which result in the scale-free nature of protein-protein interaction networks [48].

Comparison of the general amino acid composition of the CaM binding interface sequences designed for interaction with one or more targets provides valuable insight into the evolutionary processes resulting in the contemporary CaM sequence. For example, Met residues, so abundant in the CaM binding interface, were frequently postulated to be key to its ability to interact with multiple targets. Met possesses a long and flexible side chain that can, in principle, adjust for interaction with any target [49],[50]. In agreement with these observations, we show that the methionine content increases as we introduce additional interaction partners in our design procedure (Figure 9). We found a number of similar cases where the progression from single-state to multistate design converges on a sequence composition more similar to that of the evolutionary profile. For instance, the reduction in Arg content in multistate designs might result from the need for CaM to satisfy salt-bridge interactions with a number of targets. These targets show different, yet mostly positive, charge distributions; hence an Arg would be more difficult to place without destabilizing one of the CaM-target complexes. The reduction in aromatic residue content might be due to the fact that these residues need to fit in the hydrophobic pockets between CaM and the target. Since such pockets could be located in different places for the different CaM-target complexes, it would thus be difficult to provide sufficient space for aromatic amino acids in all contexts. In such cases, the compromise sequences might replace the aromatic amino acids with hydrophobic residues, such as Leu, Met, or Val, whose content increased in the transition to multistate design.

### Lessons for redesign of multispecific proteins

The results of our computational design experiments on CaM can provide useful strategies for the experimental redesign of any multispecific protein [47]. To improve the affinity of a promiscuous protein to a particular target, we should not touch the affinity-defining positions, since these positions are already highly optimized and attempts to improve them are likely to fail. On the contrary, the specificity-defining positions in multispecific proteins are usually occupied by non-optimal amino acids. For proteins that undergo a large conformational change upon binding, energetic improvements in the intramolecular interactions at these positions (Figure 5D) should result in enhanced affinity by stabilization of the protein in the target-bound conformation [28],[51]. Improvement of the intramolecular energies, however, is not likely to bring about an increase in binding specificity if interactions with different targets are conveyed through the same binding mode [52]. Optimizing the charged positions for a particular target, on the contrary, is bound to increase the protein binding specificity. Such optimization was previously used to drive the correct assembly of 4-helix bundles [53] and to substantially increase CaM binding specificity [28]. In addition, proper placement of charged residues is likely to be used by proteins to prevent folding into non-native structures [54] and to determine substrate specificity for enzymes [36].

The energetic analysis of all of the designed sequences (Figure 10), in the context of the sixteen CaM-target complex structures, helps to explain our previous experimental results on substantially increasing CaM binding specificity [17],[28]. In these experiments, we optimized CaM for interaction with a single target without incorporating an explicit negative design procedure, i.e., considering CaM interactions with alternative, undesirable targets. Unexpectedly, in the majority of cases we observed a significant decrease in CaM affinity to these other targets. There has been some controversy if one should consider negative design when designing a protein to be compatible with certain conformations [17], [54]–[59], since, as a designer, one wants to prevent the constructed sequence from folding into an alternative conformation. Our present analysis (Figure 10B , top) shows that the optimization of twenty CaM binding interface residues for a particular target is sufficient for substantially increasing (worsening) the interaction energy with other targets. Nevertheless, the necessity of incorporation of negative design is highly dependent on the problem [47]; optimizing a large number of residues and considering more dissimilar states increases the chances that positive design will suffice.

**Figure 11:**
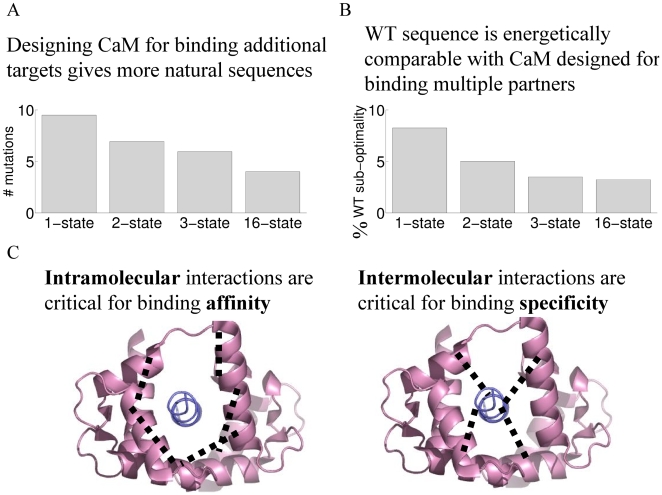
Summary of results. (A) Designing CaM for binding an increasing number of partners progressively yields more native-like sequences. (B) The WT sequence has binding energies most similar to those of CaM sequences designed for multiple interactions. (C) We find that intramolecular interactions are critical for binding affinity, whereas intermolecular interactions determine specificity toward the various targets.

In conclusion, our simulations give valuable insights as to how a prototypical multispecific protein, CaM, has evolved in nature to recognize a large number of binding partners. We uncovered both sequence and energetic tradeoffs that are imposed by multispecificity. Specifically, as additional CaM targets were explicitly incorporated in the design procedure, the resulting sequences were more similar to the native sequence (Figure 11A). Conversely, the energies with which these sequences bind the targets most closely resemble that of the WT sequence (Figure 11B). These compromises are likely to represent authentic trends in the evolution of proteins with a large number of binding partners. Our analysis also uncovered two classes of CaM interface positions: the affinity-determining positions, which stabilize the intramolecular interactions; and the specificity-determining positions, which interact strongly (but distinctly) with the various targets (Figure 11C). Our computational results will help in guiding future experiments on the redesign of CaM and other multispecific binders. Additional biochemical and structural studies of promiscuous proteins should be used to validate our findings and provide greater detail about the mechanisms employed by these proteins in achieving their diverse biological functions.

## Methods

### CaM structures and multispecific design

A thorough search of the PDB revealed 24 solved structures of CaM-target complexes. Of these, 16 were of high resolution (less than 2.5 Å for X-ray structures) and exhibited the conventional CaM-target binding mode (Figure 1A). For each structure, the interface positions were determined as those that are within 4 Å of the respective target peptide. The CaM positions found in the interface for at least 75% of the 16 structures were defined as the common binding interface, 20 in total: 11, 14, 15, 18, 19, 36, 39, 41, 51, 71, 72, 84, 87, 88, 92, 109, 112, 124, 144, and 145. All CaM structures were drawn using PyMOL [60], and sequence logos were generated using TeXshade [61] and WebLogo [62].

For the multispecific design, the goal was to predict the 100 CaM interface sequences that minimize the sum of total energies in the target structures of the respective design scenario (i.e., 1-, 2-, 3-, or 16-state designs). Thus, there were 16 single-state designs (one for each CaM-target interaction), 120 two-state design scenarios (one for each pair of the 16 CaM-target interactions), 560 three-state designs (one for each threesome of interactions), and one design of all sixteen states; this yielded 697 design scenarios in all. In each design scenario, the energies of the multiple states were uniformly weighted; for full details, see [7]. For all energy calculations, we used the ORBIT protein design force field [21] with the parameters previously used for redesign of CaM-target interactions [17]. In all subsequent design calculations, all positions were allowed to mutate to all 20 amino acids except cysteine and proline. In addition, for all structures, the peptides were allowed to vary their side chain conformations. Amino acid rotamers were defined based on the backbone-dependent rotamer library of Dunbrack and Karplus [63], with sub-rotamers added at 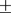 one standard deviation around the mean 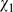 value; native sequence rotamers were included as well.

We used a combined algorithmic strategy for finding the lowest energy sequences, employing the tBMMF algorithm [7],[64] and the HERO module of ORBIT [22] and then extracting the best hundred sequences from their aggregated output. Briefly, the tBMMF algorithm provides a framework for predicting successive low energy sequences compatible with multiple protein structures. Firstly, a probabilistic graphical model is built that simultaneously models multiple protein structures of the same molecule (by requiring that the sequences predicted for the multiple structures be identical). Then, tBMMF iteratively performs energy minimization (using max-product belief propagation) within a particular sub-space of amino acid sequences in order to find the next lowest energy sequence. It then partitions this sub-space into two sub-spaces, such that subsequent low energy sequences can be readily determined; for full details, see [7],[64]. Note that only the tBMMF algorithm was capable of efficiently handling the 560 three-state designs. For the single case of 16-state design, tBMMF did not converge or yield reliable results. Therefore, the search over the sequence space was performed using a Monte Carlo simulated annealing (MCSA) algorithm [23]; at each step, a sequence was evaluated in each of the 16 complexes by calculating its minimal conformational energy using belief propagation [65]. This MCSA algorithm was repeated 10 times, for 2000 sequence steps each, and the 100 top-scoring sequences were extracted. Although we have previously shown that MCSA is often less successful at finding low energy sequences than the tBMMF algorithm [64], it seems to have performed reasonably well in this case.

### Native sequence and evolutionary profiles

The native interface sequence was extracted from the CaM structures. Evolutionary profiles were obtained by downloading and parsing the homologous sequence hits from the HSSP (Homology-derived Secondary Structure of Proteins) database [66] for each of the 16 structures and concatenating these profiles, yielding over 2100 homologues for the 20 CaM interface positions.

### Jensen-Shannon divergence (JSD) for measuring similarity between profiles

To quantitatively compare amino acid probability distributions (for a particular design position), we use the symmetric Jensen-Shannon divergence (JSD). JSD, or dissimilarity scores, were used to measure correlation either between design results and HSSP (e.g., Figure 2) or between various design scenarios (e.g., Figure 5). The JSD score ranges from 0 (identical) to 1 (“distant” distributions), so that *lower* JSD scores reflect *higher* similarity between distributions [7],[67]. The JSD between distributions 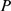 and 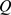 is given by:
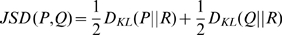
(1)where 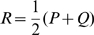 is the average distribution, and
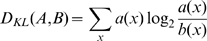
(2)is the Kullback-Leibler divergence between distributions 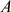 and 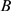. In all cases (except where noted), the mean JSD from the evolutionary profile (HSSP) for a particular CaM-target complex was calculated by averaging the JSD from the HSSP profile for all 20 interface positions.

### Prediction of residues important for affinity and specificity

To delineate CaM interface positions critical for either target affinity or target specificity, we compared the best sequences designed for interactions with the 16 single targets. This was done by calculating the JSD dissimilarity score between all 120 pairs of the 16 single-state designs at each of the design positions. Positions for which at least 50% of the pairs have a JSD dissimilarity 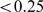 were defined as affinity-determining, and those where at least 50% of the pairs have a JSD dissimilarity 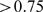 were labeled specificity-determining. For each CaM position, the results shown (Figure 5A) are for those pairs of structures for which the position interacts with the target in both structures; results were similar when considering all pairs of structures (not shown). Per-position energy contributions (e.g., Figure 5D,E) were calculated using the EANAL module of the ORBIT program.

### Quantifying multistate sequence compromise

For a particular 2-state design scenario, the profile based on its 100 lowest energy sequences was compared to those designed for interactions with the same two single targets (1-state designs). The comparison was performed at each of the 20 design positions. For each position, the multistate sequence compromise was categorized (Figure 7) based on a JSD comparison between the two 1-state designs and between the same 1-state designs and the 2-state design. We defined 5 intuitive categories: “Kept same” - the 1-state designs predicted similar results (pairwise JSD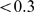) and the 2-state design was similar to both of them (both pairwise JSD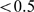); “Combined” - the 1-state designs were dissimilar (pairwise JSD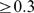), but the 2-state design was similar to both of them (JSD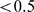); “Preferred one” - 2-state design was similar to only one of the 1-state designs (JSD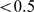); “New aa” - the 1-state designs predicted dissimilar results (pairwise JSD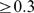) and the 2-state design was different from both of them (both pairwise JSD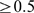); “despite same” - despite the 1-state designs predicting similar results (pairwise JSD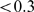), the 2-state design was different from both of them (JSD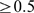).

For positions where the 2-state design “preferred one” of the 1-state designs or chose a new profile altogether (“New aa”, “despite same”), we quantified to what degree this affected the biological quality of the sequence results (Figure 8). To do this, we first calculated the per-position JSD scores comparing the 2-state profile to HSSP. Then, we constructed the profile resulting from averaging the two 1-state design profiles and calculated its per-position JSD scores from HSSP. For a particular position, the difference between these JSD values (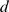) was used to define the effect of multistate compromise: “No Change” - 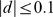; “Benefit” - 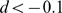; “Loss” - 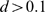. Recall that lower JSD scores from HSSP indicate greater similarity to the evolutionary profile, so that a decrease in JSD is termed beneficial. We chose to represent the performance of the two 1-state design scenarios using their average profile since, barring any external information, the most logical procedure would be to simply combine these two profiles as a proxy to the low energy sequence space compatible with both targets. For all calculations, we show results for those pairs of structures for which the position interacts with the target in both structures; results were similar when considering all pairs of structures.

### Energetic compromise for multistate design

To characterize the tradeoff in energetic stability required for promiscuity, we quantified the changes in sequence energy resulting from the inclusion or exclusion of additional target states in the multispecific design procedure (Figure 10). Recall that the design results in this paper are based on the 100 lowest energy sequences for each of the 697 design scenarios detailed above, yielding a total of 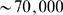 sequences. Firstly, we calculated the energy of each of these sequences in each of the 16 target structures (over 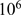 calculations in total). To efficiently perform these calculations, we utilized belief propagation (and Monte Carlo simulated annealing if the belief propagation algorithm did not converge, see [64]) to calculate the lowest energy rotamer conformation of each such sequence threaded onto the structure of each CaM-target complex. For each structure, the energy of a particular sequence was normalized by the absolute value of the energy of the best sequence designed for that structure. Then, for each combination of design scenario and structure, we chose the sequence with lowest normalized energy when threaded onto the structure, among the 100 sequences designed for that scenario. This yielded the final 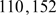 normalized energies (corresponding to 697 sequences
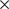
16 structures) utilized for plotting Figure 10 and Figure 11C.

Now, denote by A, B, C, and D any four arbitrary CaM states, i.e., complexes of CaM with different targets. For all 12 histograms in Figure 10 (3 rows
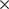
4 columns), we enumerate all possible choices of the corresponding CaM states. For each such choice, we calculated the designated differences in normalized energy, and all resulting values were plotted in the respective histogram. For example, in the bottom panel of Figure 10 (row 3, column 2), consider each of the 16 CaM-target complexes as state A. Then, consider all triples of other possible states as B+C+D. Finally, calculate the difference in normalized energy in state A, between the sequence resulting from the simultaneous design of B, C, and D and the sequence resulting from the exclusive design of A. This difference, necessarily positive, was one of the 7280 values (16 choices for A 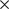
455 choices for B+C+D) used in creating this frequency histogram.
